# Comment on “Bioaccumulation of Methyl Siloxanes in Common Carp (*Cyprinus carpio*) and in an Estuarine Food Web in Northeastern China”

**DOI:** 10.1007/s00244-019-00681-2

**Published:** 2019-12-14

**Authors:** Jaeshin Kim, Kent Woodburn, Katie Coady, Shihe Xu, Jeremy Durham, Rita Seston

**Affiliations:** 1 Toxicology and Environmental Research and Consulting, The Dow Chemical Company, Midland, MI USA; 2 Hyla Environmental Consulting, LLC, Midland, MI USA

## Abstract

We have reviewed a paper titled “Bioaccumulation of Methyl Siloxanes in Common Carp (*Cyprinus carpio*) and in an Estuarine Food Web in Northeastern China” by Xue et al., which was published in the *Archives of Environmental Contamination and Toxicology* in 2019. In the paper, the authors presented and discussed the measured bioconcentration factors (BCFs), biomagnification factors (BMFs), and trophic magnification factors (TMFs) of selected volatile methylsiloxanes in Shuangtaizi estuary in China. Although we appreciate their efforts for sample collection and data analysis, we have identified significant errors in calculations of BCFs, TMFs, and BMFs, as well as animal welfare issues and food web trophic level assumptions. Based on the data, we have attempted to correct some of the analysis and offered a more complete and robust interpretation of the related data, when possible. Collectively, these errors would likely lead to very different conclusions than yours in the paper.

## Summary of Xue et al. (2019)

Xue et al. (2019) discussed bioaccumulation of volatile methylsiloxanes (VMS) in Shuangtaizi estuary located in the northeastern region of Bohai Sea, China (Xue et al. 2019). They determined three metrics to assess bioaccumulation: bioconcentration factor (BCF), biomagnification factor (BMF), and trophic magnification factor (TMF) for selected VMS. BCFs were measured based on an exposure test of common carp (*Cyprinus carpio*) for 32 days followed by 32 days of the depuration period. BMFs and TMFs were measured from biota samples that were collected in Shuangtaizi estuary. These collected samples included three different species of fish, eight different invertebrate species, and plankton.

Based on the measurement and data analysis, the authors concluded that:

- Octamethylcyclotetrasiloxane (D4) had strong bioconcentration potential (with a BCF of 6197 L kg^−1^),
- Decamethylcyclopentasiloxane (D5), dodecamethylcyclohexasiloxane (D6), and linear siloxanes (L7–10) were trophically magnified (with TMFs much > 1) in a food chain, and
- D4–D6 and L7–L10 were biomagnified from planktons to Japanese snapping shrimp (with BMFs > 1).

In addition, the authors claimed that this is the first study to investigate the bioaccumulation behaviors of methyl siloxanes by coupling BCF and BMF with TMF.

## Critical Reviews

Although the paper provided details of analytical methods and quality assurance and quality control (QA/QC) procedures, there are significant scientific errors in data analysis and biased selections of datasets to support their hypothesis of significant bioaccumulation of VMS.

### Measurement of BCFs

**Fig. 1:**
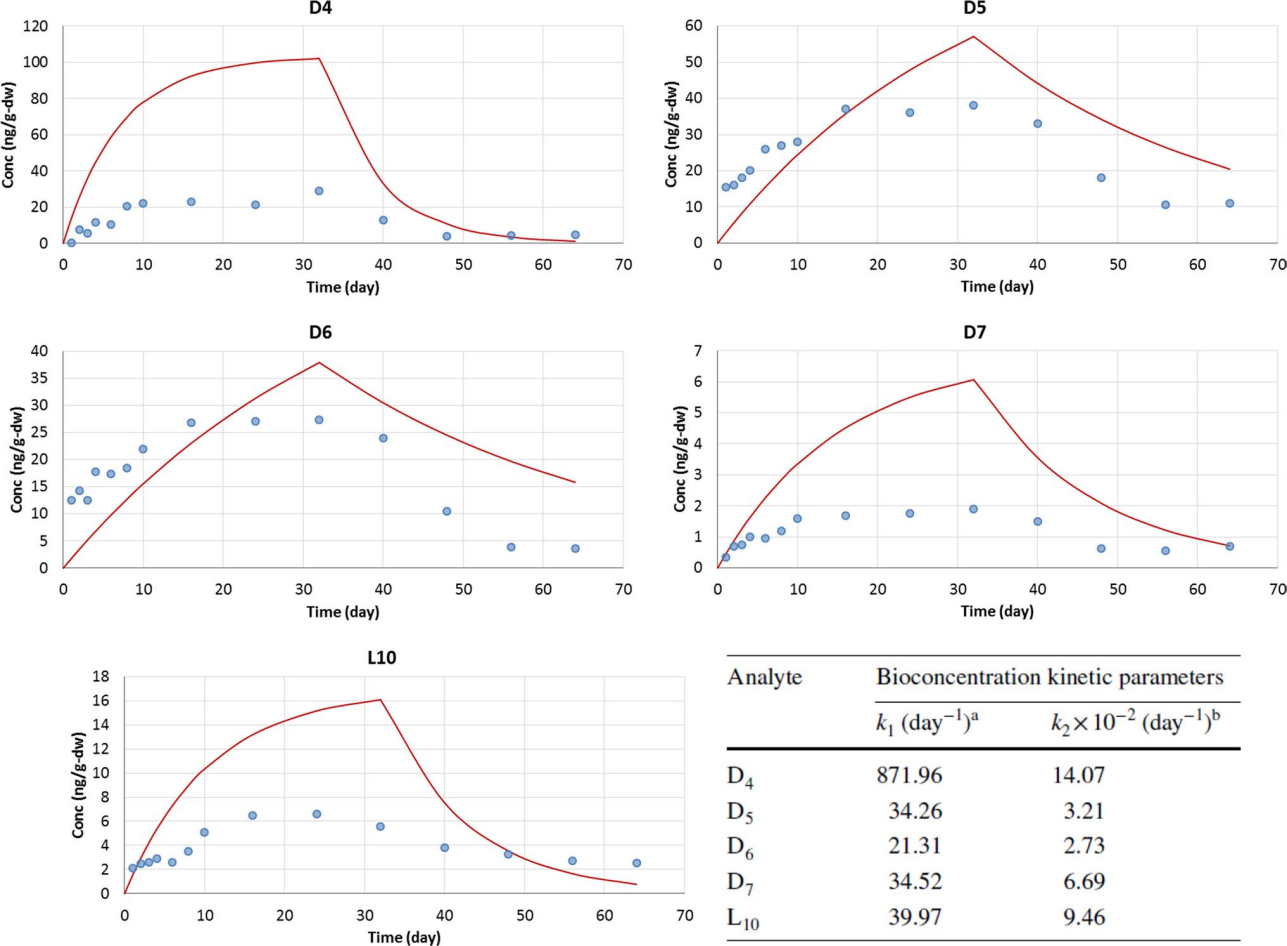
Concentrations of D4, D5, D6, D7, and L10 (ng g-dw^−1^) during the periods of exposure (0–32 days) and depuration (32–64 days). The blue circles are measured biota concentration over time. The red lines are estimations of concentration during each period (i.e., Equation 2 for exposure and Eq. 5 for depuration). Values of *k*_1_ and *k*_2_, shown in the bottom right, are from Table 1 of Xue et al. (2019). To convert dry-weight basis concentration to wet-weight basis concentration, the water content of the common carp was assumed to be 70%

The result of the exposure test with common carp is shown in Fig. 1 of Xue et al. (2019) with dry-weight (ng g-dw^−1^) biota concentrations of D4, D5, D6, D7, and L10 measured in muscle tissue over the duration of the study. The calculated uptake and clearance rates (i.e., *k*_1_ and *k*_2_, respectively) are shown in Table 1 of Xue et al. (2019). These rates and are based on regression curves using the equation *C*_*F*(*t*)_ = (*k*_1_/*k*_2_) *C*_*w*_ [1 − exp(− *k*_2_*t*)] for the exposure period [Eq. 2 and Fig. 3 in Xue et al. (2019)] and the equation *C*_*F*_ = *C*_*F*0_ exp (− *k*_2_*t*) for the depuration period [Eq. 5 and Fig. 4 in Xue et al. (2019)]. We reproduced the regression results using the calculated values of *k*_1_ and *k*_2_ in the specified equations above and plotted measured biota concentrations and the regression curves (Fig. 1).

These plots clearly showed that the equations used to calculate update and depuration rates are not well-aligned with measured biota data. Furthermore, the lines in Fig. 3 of Xue et al. (2019) showing uptake do not start with the origin (*C*_*F*_ = 0 at *t* = 0 in Eq. 2). This indicates that values of *k*_1_ and *k*_2_ in Table 1 are not strictly from the specified Eqs. 2 and 5 of Xue et al. (2019). In addition, there may be some contamination of the test fish according to the measurements where the test materials are detected in biota at an early phase of the exposure (i.e., Day 1). However, this issue was not discussed in the publication.

**Table 1:** Bioconcentration kinetic parameters of D4, D5, D6, D7, and L10 in common carp

	Simultaneous fit: first-order Berkeley-Madonna modeling				Sequential fit: first-order elimination fit	
	*k*_1_ (L kg-ww^−1^ d^−1^)	*k*_2_ (d^−1^)	BCF (L kg-ww^−1^)	Half-life (d)	*k*_2_ (d^−1^)	Half-life (d)
D4	184	0.11	1673	6	0.06	12
D5	47.5	0.076	625	9	0.05	14
D6	40.1	0.095	422	7	0.073	9
D7	13.9	0.075	185	9	0.037	19
L10	14.3	0.068	210	10	0.024	29

Parameters *k*_1_ (uptake rate constant) and *k*_2_ (elimination rate constant) were optimized via first-order Berkeley Madonna modeling of uptake and elimination (simultaneous fit) and simple first-order sequential fit of the elimination data

**Fig. 2:**
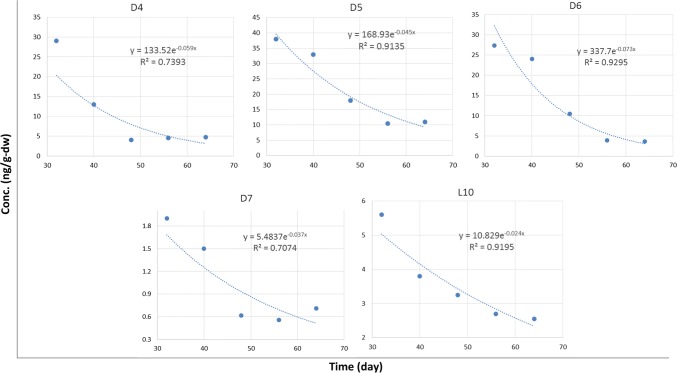
Concentrations of D4, D5, D6, D7, and L10 (ng/g-dw) during the depuration period (32–64 days). The blue circles are measured biota concentration over time. The blue dotted lines are exponential regression curves based on Eq. 5 of Xue et al. (2019)

Plots in Fig. 4 of Xue et al. (2019) during the depuration phase were based on Eq. 5 that is a simple exponential decay function. However, the red lines in the plots are not such exponential function. The following plots are reconstructed using Eq. 5 optimized for the measured biota concentrations. These reproductions are not consistent with plots of Fig. 4. There is also an inconsistent data point. In Figs. 1d and 3d of Xue et al. (2019), D7 concentration at Day 32 is 1.9 ng g-dw^−1^, but it is 1.7 ng g-dw^−1^ in Fig. 4d (Fig. 2).

**Fig. 3:**
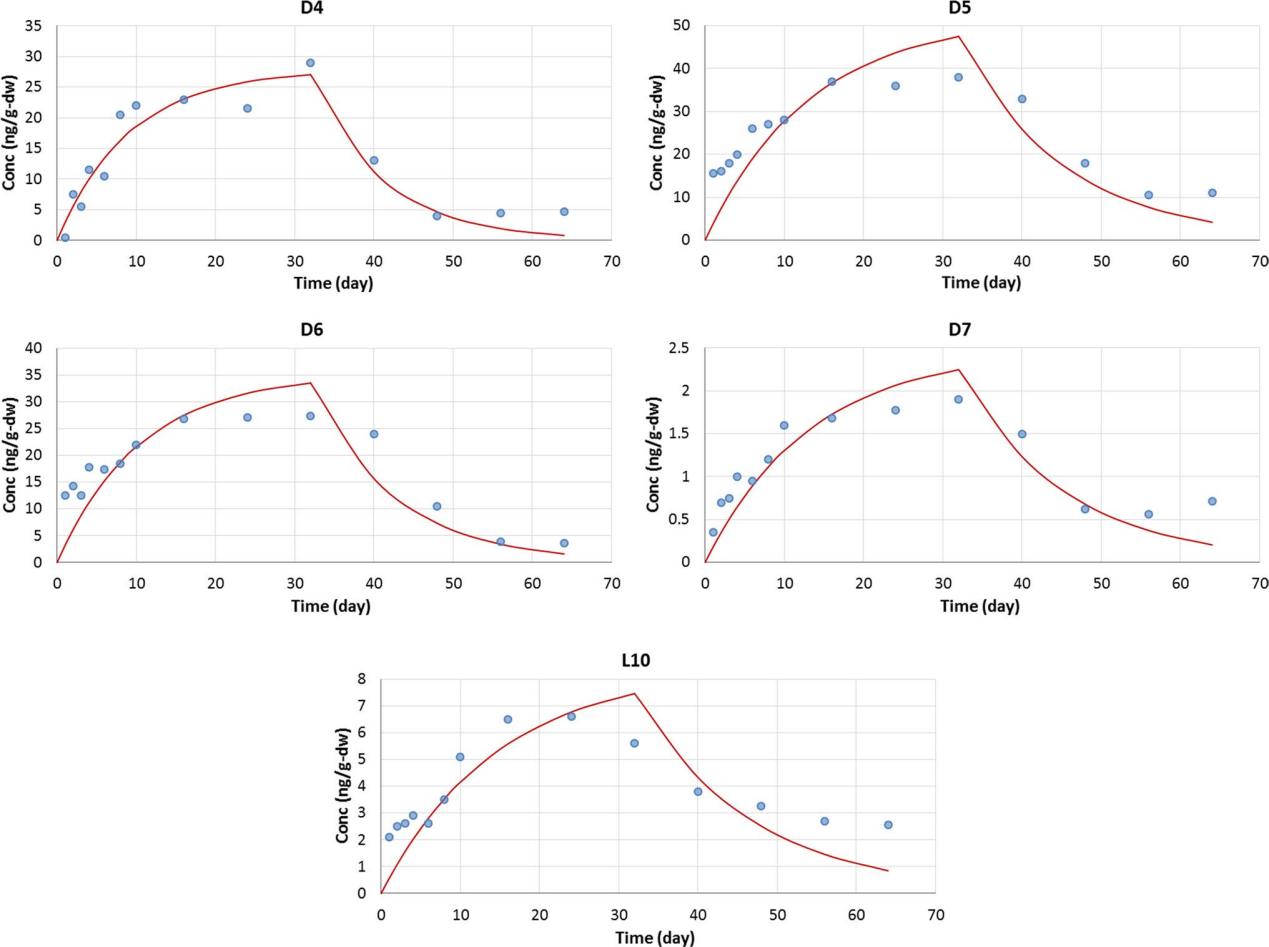
Concentrations of D4, D5, D6, D7, and L10 (ng g-dw^−1^) during the periods of exposure (0–32 days) and depuration (32–64 days). The blue circles are measured biota concentration over time. The red lines are estimations of concentration during each period (i.e., Equation 2 for exposure and Eq. 5 for depuration). Values of *k*_1_ and *k*_2_ are from the new regression using the Berkeley Madonna program (Table 1). To convert dry-weight basis concentration to wet-weight basis concentration, the water content of common carp was assumed to be 70%

Because we found errors in the regression for the biota data in the publication, we also determined values of *k*_1_ and *k*_2_ by optimizing the parameters in Eqs. 2 and 5 via first-order Berkeley Madonna modeling of uptake and elimination (simultaneous fit) and simple first-order sequential fit of the elimination data (Berkeley Madonna: Modeling and Analysis of Dynamic Systems Version 8.3.18, https://berkeley-madonna.myshopify.com/). Note that the paper provided concentration information on a dry-weight basis and reported BCF values in wet-weight basis. Because no water content (or solid content) of the fish is known, 70% of water content (or 30% of solid content) was assumed. The results of optimized parameters from the new regression are shown in Table 1. The plots of measured biota concentration (in blue circles) and optimized uptake and depuration curves over time also are shown in Fig. 3. The plots show that the new regression curves are fitted to Eq. 2 and Eq. 5 much better than those of Xue et al. (2019) in Fig. 1. The new values of *k*_1_ and *k*_2_ are different from those of Xue et al. (2019), and the new BCF values are less than 2000 L kg-ww^−1^ for all VMS tested. Therefore, data analysis of the exposure test conducted by Xue et al. (2019) is likely incorrect and the BCF values cannot be accepted. Instead, a proper data analysis would support much lower BCF values for D4, D5, D6, D7, and L10 in common carp than those of Xue et al. (2019).

An additional factor in the carp BCF studies is the fact that the fish apparently were not fed during the 64-day experiments, which amounts to animal abuse in this case and resulted in the loss of body weight for the fish under study during the greater than 2-month testing period. Table S1 of Xue et al. (2019) shows significant decrease in body weight especially in the later phase of the test. However, OECD (2012) Guidelines 305 states in Item 45, “During the acclimation and test periods, to feed an appropriate diet of known lipid and total protein content to the fish in an amount sufficient to keep them in a healthy condition and to maintain body weight.” All chronic assays, including fish BCF studies, conducted under U.S., Canadian, or European test guidelines require the feeding of fish for studies lasting longer than 4 days. It is entirely possible that this long time period (64 days) without food could have altered uptake, elimination, and metabolic responses of the test species exposed to the cyclic and linear siloxane materials, thereby altering the BCF values produced during the experimental period.

Finally, the carp concentrations presented by Xue et al. (2019) are corrected for fish dry weight (dw) not fish wet weight (ww), which is the basis used for global regulatory expression of bioaccumulation (B) metrics. The laboratory method promulgated by the European Union for evaluation of fish BCF, OECD (2012) Guidelines 305 is based on use of wet weight or lipid weight concentration data and its interpretation for calculation of a guideline fish BCF value. Thus we suggest that the authors specify the units that used for the outcomes of the kinetic parameters and BCF values.

### Measurement of TMFs

Table 2 of Xue et al. (2019) compiles all the field biota concentrations of D4–D7 and L7–L10 on both a wet- and lipid-weight basis. Based on the data, TMFs were calculated based on the regression described in Eq. 8 and Eq. 9 of Xue et al. (2019). The plots of log-transformed biota concentrations versus trophic level (TL) are shown in Fig. 6, where only D5, D6, L7, L8, L9, and L10 are shown (not D4 or D7).

However, it should be noted that the plots only reflected one food chain in the food web (see Fig. 5 of Xue et al. 2019), more specifically: planktons (TL = 2.14) → ark shell (TL = 2.78) → Neverita’s albumen (TL = 2.95) → Chinese ditch prawn (TL = 2.98). The paper failed to provide TMF values based on other food chains and all these measurements. Furthermore, the single food chain that was examined for deriving TMF values was based on small sample sizes of biota (e.g., *n* = 1 for ark shell and *n* = 2 for Chinese ditch prawn).

Because the real food-web in the test area is complex by nature, it is highly unlikely the simple food chain would represent environmental reality, and the small sample sizes of some of the biota in the examined food chain are too small to account for expected environmental variability (Borgå et al. 2012). Additionally, as the authors acknowledge, the enrichment relationship between the two isotopes (^13^C and ^15^N) was not strong, and a proper food chain could not be established with any confidence. In particular, the ^15^N values that are typically used to calculate TL values (see Table S5 and Figure S2 of Xue et al. 2019) are all statistically equivalent values, with the exception of the “planktons” ^15^N value. Generally in aquatic food webs, the enrichment of ^15^N values from one trophic level to the next is approximately 2–5‰, with a generic value of ^15^N enrichment of 3.4‰ per trophic level step recommended for constructing food webs without a priori knowledge of the system (Deniro and Epstein 1981; Jardine et al. 2006; Minagawa and Wada 1984; Post 2002). Excluding the ^15^N value for “planktons,” this level of ^15^N enrichment is entirely lacking in this aquatic system located in northeastern China. These data do not support that the samples taken for this study are reflective of an existing food chain; therefore, calculation of TMF values from these samples is not supported as environmentally relevant.

In addition, regarding the specimen masses in the food chain, the prey–predator relationship in the food chain is unusual. The only ark shell collected was an individual with a mass of 71 g (not clear if this was with or without shell), the majority of the species which is indicated to prey upon the ark shell, Neverita albumen, a predatory snail, had masses ranging from 2.1 to 4.7 g, with one individual of 17.9 g. The “apex predator” of this food chain is the Chinese ditch prawn with specimen masses ranging from 1.0 to 3.7 g. Although the ark shell → Neverita’s albumen → Chinese ditch prawn may be a feasible food chain in this ecosystem, it seems highly unlikely that the individual specimens collected and analyzed here are reflective of that food chain. Individual size and life stage need to also be taken into consideration.

Thus it is strongly recommended to use all the data for TMF calculation because the real food-web is intrinsically incorporated in the assessment. Kidd et al. (2018) also suggested key principles for evaluation of TMF studies including a minimum food web TL difference of 2.0 and balance in the number of samples for low and high trophic level species. Using all the data in Table 2 of Xue et al. (2019), TMF were calculated as shown in Table 2 below [with TMFs of Xue et al. (2019) in the last column]. Plots of biota concentrations versus trophic level are shown in “Appendix 1” of this document.

**Table 2:** Recalculation of trophic magnification factors (TMFs) using all the biota data of Shuangtaizi estuary, China, in Table 2 of Xue et al. (2019)

VMS	Without plankton			With plankton			
	TMF	*R*^2^	*p* val	TMF	*R*^2^	*p* val	TMF (Xue et al. 2019)
D4	0.081	0.4701	1.99E−02	0.459	0.1134	2.85E−01	Not provided
D5	0.137	0.3031	7.93E−02	0.411	0.1530	2.09E−01	11.0
D6	0.051	0.3292	6.50E−02	0.480	0.0492	4.88E−01	7.1
D7	0.041	0.3491	5.56E−02	0.378	0.0816	3.68E−01	Not provided
L7	0.033	0.3544	5.33E−02	0.664	0.0116	7.39E−01	10.0
L8	0.198	0.2859	9.01E−02	1.026	0.0002	9.70E−01	6.6
L9	0.461	0.0364	5.74E−01	1.828	0.0463	5.02E−01	5.3
L10	0.959	0.0001	9.77E−01	1.783	0.0445	5.10E−01	3.0

As shown in Table 2, the plots of all the lipid-weight concentrations show trophic dilution (TMF < 1) for D4, D5, D6, D7, and L7, whereas L8-L10 indicated TMF values above one. Especially when biota concentrations without plankton were used, the trends were improved (larger *R*^2^ and smaller *p* values) and all VMS have TMF < 1 (from 0.05 to 0.96).

Because the authors presented TMFs based on the second food chain (FC-02) out of three, it would be appropriate to test the other food chains (FC-01 and FC-03) to examine the complete data set, rather than only assessing one potential food chain. TMFs for each food chain were calculated and shown below. The results clearly show that the authors “cherry-picked” the second food chain to demonstrate trophic magnifications of VMS. Although the authors’ TMF values should be relevant to TMFs of FC-02, we cannot reproduce their calculations exactly. One reason might be that we used average values for each species provided in Table 2 of Xue et al. (2019), whereas authors might use individual data points. Additional plots of lipid-weight concentrations versus trophic level are shown in “Appendix 2” of this document (Table 3).

**Table 3:** Trophic magnification factors (TMFs) calculated for each food chain established by Xue et al. (2019)

	TMF (with plankton)			
	FC-01	FC-02	FC-03	FC-02 (Xue et al. 2019)
D4	0.41	2.77	0.62	Not provided
D5	0.47	1.53	0.46	11.0
D6	0.48	5.25	0.62	7.1
D7	0.37	4.06	0.51	Not provided
L7	0.92	13.03	0.90	10.0
L8	1.26	4.13	1.28	6.6
L9	3.53	5.82	1.75	5.3
L10	1.71	3.49	1.99	3.0

The results clearly showed that TMFs of D4, D5, D6, D7, and L7 are less than 1 for FC-01 and FC-03, although they are greater than 1 for FC-02. Given that the biota plots of all data points (rather than individual food chain) are known, conclusions based on only FC-02 cannot be accepted without providing obvious reasons to exclude TMF values for FC-01 and FC-03. Again, because there are significant uncertainties around isotopic ratios, representative species (limited sample sizes and narrow range of trophic level), and the food chains proposed by Xue et al. (2019), it is recommended to calculate TMFs incorporating uncertainties of food chains and to include all the measurement of biota concentrations which are the outcomes of complex food-webs.

### Measurement of BMFs

Authors calculated BMFs of VMS from planktons (TL 2.14) to Japanese snapping shrimp (TL 2.59) using Eq. 10 in Xue et al. (2019). The calculated BMFs were greater than 1 for D4–D6 and L7–L10, indicating biomagnification from the prey–predator relationship. These BMF values were not able to be completely reproduced; relatively large differences between the two calculations were observed especially for D5, D6, L7, and L8 (see the table below).

**Table:** 

Species	TL	D4	D5	D6	D7	L7	L8	L9	L10
Concentration (ng g-lw^−1^)									
Japanese snapping shrimp	2.59	750	181	612	364	520	113	108	99.4
Planktons	2.14	239	156	141	103	60.1	30.2	30.2	39.3
BMF									
Xue et al. (2019)		3.2	5.2	9.0	–^a^	11	9.2	3.4	2.2
New calculation		2.59	0.96	3.59	2.92	7.15	3.09	2.95	2.09

^a^Not provided

The authors evaluated BMFs from just one prey–predator relationship, but not many other relationships in the food-web in Shuangtaizi estuary. Since TMF is basically an average of BMFs of all prey–predator relationships, it is expected that most BMFs are less than one for all VMS at higher trophic levels (see “Appendix 1” for biota concentrations versus trophic level without plankton). Because the authors’ knowledge of the food web is not demonstrated in the text nor does the uniformity of the ^15^N data give information concerning prey/predator relationships in the food web, the presentation of BMF values in this paper is nearly a random ratio of concentrations of VMS in various species. The authors came to a premature conclusion considering only one set of biomagnification data. It is recommended to evaluate BMFs for other prey–predation relationships with exclusion of plankton (as discussed above). This would eventually lead to a more relevant discussion on TMFs of VMS.

### Claiming the First Study of Coupling BCF and BMF with TMF

The authors claimed, “This is the first study to investigate the bioaccumulation behaviors of methyl siloxanes by coupling BCF and BMF with TMF.” In our opinion, however, “coupling” has not been conducted in the newest sense. All the TMF studies include BMF by default. Here the BCF measurement was one thing (with Common carp) and the TMF measurement was another. No same biota species were tested for both BCF and TMF to couple the two metrics. Since authors did not trace the origin of the common carp that were bought from a local market, there was no valid evidence that the fish were from the same field sampling area examined for TMF derivation.

### Analytical Concerns

The authors stated they tried to reduce the contamination of VMS in sampling, treatment and analysis stages. However, it is very unlikely they would be able to achieve the LOD in water of 0.1–1 ng L^−1^ using a DB-5 column. GC–MS analysis in samples are often plagued by elevated levels due to low bleed rubber septa and PDMS based columns such as the DB-5 used (Varaprath et al. 2000, 2006), alternatives columns and GC–MS components are available were not utilized by the researchers.

The LOD and LOQ determinations are not well defined in the paper. There is no information provided regarding the number of replicates or the concentration of internal standard that was spiked to all samples. The authors state that procedural blanks were done with every 10 samples, however Table S4 shows blanks 1–14 only for biota samples (indicated by the unit, ng/g-ww in the caption) but not for water samples. Additionally, the blanks were presented as a concentration, but typically procedure blanks are presented as an amount.

### Lack of VMS Concentrations in Sediment and Water

VMS concentration in sediment in Bohai Sea has a strong gradient along the coastlines, which indicates that the exposure levels to biota species are different from location to location (Zhi et al. 2019). It is strongly recommended to measure VMS concentrations in sediment and water to evaluate the degree of exposure of benthic invertebrates and fish for different sampling locations. Zhi et al. (2019) concluded that all cyclic and linear VMS (D4–D6 and L5–L16) in benthic mollusks sampled from seven different locations in Bohai Sea, China showed biota-sediment accumulation factors (BSAF) much less than 1.7, indicating less potential bioaccumulation for VMS.

### Minor Typos

- Joyner’s tonguesole (*Arelicus joyneri gunther*) in Section “Food Web Sample Collection”: gunther is not a genus nor species. Günther is a researcher who studied this species. Correction: joyner’s tonguesole (*Arelicus joyneri*).
- The unit of *k*_1_ in Table 1 is day^−1^. It should be L kg-ww^−1^ day^−1^.
- In Table S2, the unit of body length of biota samples is cm. It should be mm.

## Conclusions

We have reviewed the data, methods, and overall analysis of the paper published by Xue et al. (2019). There are clear errors in study conduct and standardization of data and measurements, animal welfare issues, food web trophic level assumptions, sample collection issues, and incorrect calculations of BCFs, TMFs, and BMFs. Collectively, these errors in collection, analysis, and judgment would likely lead to very different conclusions than those offered by the authors in the publication. In this analysis, we have attempted to correct some of the analysis and offer a more complete and robust interpretation of some of the data, when possible.

The siloxane BCF values were recalculated using OECD standard methods and all tested siloxane values were < 2000 L kg-ww^−1^, indicating that none of the materials would be considered “B” per common regulatory guidance. The reanalyzed TMF values (without plankton) were all less than 1, confirming using field data that VMS did not exhibit biomagnification via the food web for Shuangtaizi estuary in China.

Based on our careful review, the paper contains significant errors in data analysis and inconsistent data interpretation as well as erroneous conclusions. Thus, we suggest the editors of *Archives of Environmental Contamination and Toxicology* request the authors to withdraw the publication from *Archives of Environmental Contamination and Toxicology*, although we respect the editor’s initial decision.
